# Female gender and Helicobacter pylori infection, the most important predisposition factors in a cohort of gastric cancer: A longitudinal study

**Published:** 2016

**Authors:** Shahram Agah, Hossein Khedmat, Mohammad Ebrahim Ghamar-Chehred, Reza Hadi, Aghdas Aghaei

**Affiliations:** 1 Colorectal Research Center; Iran University of Medical Sciences, Tehran.; 2 Baqiyatallah Research Center for Gastroenterology and Liver Disease; Baqiyatallah University of Medical Sciences, Tehran, Iran.

**Keywords:** Helicobacter pylori, Gender, Female, Stomach cancer, Gastric carcinoma, Iran

## Abstract

**Background:**

Gastric cancer (GC) is one of the most common Cancers in the world and Helicobacter pylori (HP) infection is considered a causative factor. The aim of this study was to determine the characteristics and the associated factors of (GC) in a small cohort.

**Methods:**

Overall, 54 patients with diagnosed gastric cancer were followed-up at the Department of Gastroenterology & Hepatology at Baqiyatallah University of Medical Sciences. 37 (68.5%) of them were positive for *H pylori* infection in histopathological evaluations. Univariate and multivariate regression analyses were used to determine the associations of demographic features and HP infection status with GC characteristics and prognosis.

**Results:**

Univariate analysis showed female gender (odds ratio (OR): 6.53; 95% confidence interval (95%CI): 1.59-26.8; P=0.008), and illiteracy (compared to intermediate education; OR: 5.9, 95%CI: 1.37-25.43; p=0.05) were associated significantly with higher rate of HP infection. After a mean±SD follow-up duration of 254±329 months, only female gender was significantly associated with HP infection in GC (OR:4.56; 95% CI: 1.0-21.76; P=0.05). *H pylori* positive patients had significantly higher grade of GC (OR: 3.97; 95% CI: 1.0-16.16; P=0.05), and a trend toward greater GC stage (OR: 4.46, 95% CI: 9.39-21.23; p=0.06). There was no association between survival rate and *H pylori* infection.

**Conclusion:**

In the current study, we found a significant association of female gender with GN and an association of higher grade of GC with female gender. These findings may indicate a sex disparity in susceptibility to HP infection regarding GC future studies of larger populations are recommended.

**G**astrointestinal malignancies are the most common cancers in the world and gastric cancer is the leading cause of cancer death with an annual incidence rate of about one million a year (1). Moreover, the massive associated mortality rate makes it the second leading cause of dancer-related deaths after lung cancer (2). In Iran, national reports as well as regional studies have indicated that gastric cancer is responsible for the highest cancer incidence rate among males (3, 4). Moreover, this cancer has been reported as the leading cause of mortality with a far large gap from other neoplasms, both in Iranian males and females (5).

This is not surprising news that gastric cancers in Iran is one of the higher risk areas in the world with a relatively high associated mortality (4). Helicobacter pylori (HP) is a Gram-negative, spiral flagellate bacillus living in the gastric mucus adherent to mucosa that infects almost half the world’s population and produces a chronic infection that can lead to several disorders including gastric and duodenal ulcers, gastric cancer, and B-cell mucosa-associated lymphoid tissue lymphoma (6-8). 

This agent colonizes the human stomach and establishes a long-term infection of the gastric mucosa (9). Strong evidence suggests that HP has an overwhelming impact on the development of gastric cancers (7). Several studies have been conducted to determine the predisposition of human subjects to gastric cancer. These studies have shown contribution of different host characteristics and various types of HP in the development of gastric cancers. The aim of this study was to determine the associated factors of HP infection in patients with gastric cancer and to determine the relationship between severity of gastric cancer with HP.

## Methods

**Patients:** This study was conducted at the Department of Gastroenterology & Hepatology at Baqiyatallah University of Medical Sciences. Overall, 54 patients consecutively diagnosed with gastric cancers were followed-up for their post diagnosis course. There were 30 (55.6%) males and 24 (44.4%) females. The mean±SD age of patients was 64.3±14.9 years. Diagnosis was based on endoscopy with biopsy and histopathological evaluations, in all the patients. Surgical intervention: A rather uniform approach to the patients was followed-up by the Head department. The standard surgical approach included wide gastric resection with radical (R2) lymphadenectomy, in which the second tier of lymph nodes (N2), beyond the perigastric nodes (NI), is removed. All total gastrectomy procedures were accompanied by Roux-en-Y reconstruction with a 40-50 cm Roux loop of jejunum, to keep bile and pancreatic juices out of the esophagus and respiratory tract. 

Pathology, grading and staging: Carcinoma was diagnosed when tumors invaded the lamina propria or were through the muscularis mucosae and dysplasia was defined as histopathological evidence for neoplastic transformation of epithelial cells limited to the epithelium upon the intact basement membrane (10). Gastric biopsy specimen were fixed in buffered formalin and embedded in paraffin; then the sections were stained with haematoxylin and eosin and modified Giemsa techniques. All histological assessments were made by lecturers at the Department of Pathology of Baqiyatallah University of Medical Sciences, under the supervision of the head of the department. 

Specimen achieved from the antrum and corpus tissue were subjected to evaluation for HP existence, intensity of either polymorphonuclear or mononuclear cells infiltrates, glandular atrophy, and intestinal metaplasia as stipulated by the updated Sydney system (Houston) (11). Paraffin-embedded tissue sections were stained with hematoxylin and eosin to grade the severity of gastritis and with Giemsa stain to detect HP. Each participant was given a histologic diagnosis that represented the most advanced grade seen at different sites of biopsy. Patients with chronic nonatrophic gastritis were excluded from the study, while their lesions were not cancerous or pre-cancerous. Staging was performed according to the recommendations of the Union for International Cancer Control (UICC) (12, 13).

Statistical analysis: SPSS Version 17.0 (SPSS corp.; Chicago, Il, USA) was used for analyses. A p≤0.05 was considered significant. Chi-square test was used to evaluate associations between the study patients’ gender, blood group and Rh status, educational level, self-rated economic level, family history, endoscopic and CT-scan reports and cancer grading and staging with HP positivity status. Student’s t-test was used to evaluate the age of the study population and its potential association with HP status. Logistic regression models were used for further evaluation of the predictive value of the demographic data and cancer grading and staging with HP status, and multivariate analyses were used to evaluate the independence of the found associations. Life tables and Kaplan Meier methods have been used for defining and comparing one year survival rates in patients with and without HP infection. 

## Results

Table 1 summarizes the differences in the demographics and disease characteristics of gastric cancer regarding their histopathological findings about HP replication within their gastric lesions. Overall, 37 patients (68.5%) were positive for HP in their histopathology report. HP positive gastric cancer patients had comparable age at diagnosis with their HP negative counterparts (P=0.5). Blood groups and Rh status of gastric cancer patients were comparable between HP positive and negative patients (P=0.7 & 0.6, respectively). Self-defined financial status was also equal in the two groups (P=0.7). Univariate logistic regression models revealed female gender (Odds ratio (OR): 6.53; 95% confidence interval (95% CI): 1.59-26.8; P=0.008) and illiteracy (compared to intermediate education; OR: 5.9, 95% CI: 1.37-25.43; P=0.05) as significant associated factors of H pylori infection 

But after multivariate analysis, female gender remained the sole independent associated factor of H pylori infection positivity within gastric cancer tissue (OR: 4.56; 95% CI: 1.0-21.76; P=0.05). Regarding the disease characteristics of patients, HP positive patients had higher grade cancer (OR: 3.97; 95% CI: 1.0-16.16; P=0.05), and there was a trend towards higher disease stage in HP positive patients which did not reach to a statistically significant level (OR: 4.46, 95% CI: 9.39-21.23; P=0.06).

Patients were followed-up for a mean±SD of 254±329 months. At the end of follow up period, 25 (48%) of the patients died. Treatment modality was not different between the two study groups (P=0.8; table 2). Survival analysis showed no significant association between HP replication in gastric lesions and patients’ outcome (P=0.9). Life tables were used to define survival of gastric cancers. One year survival rate for patients’ gastric cancers positive for HP in gastric lesions was 45%, while it was 50% for HP negative patients.

**Table 1:** Associated factors to HP status of gastric lesions in our patient population

**Factors **	**HP status**		**Sig.**
	**Positive**	**Negative**	
Gender male (%)	16 (43.2)	14 (82.4)	0.009
Age (mean±SD)	15.1±2.6	66.3±14.6	0.5
Blood group (%)			
A B O AB	13(40.6) 4(12.5) 13(40.6) 2(6.3)	4(30.8) 2(15.4) 7(53.8) 0	0.68
Rh positive (%)	34 (85.7)	12 (90)	0.619
Educational levels			
Illiterate (%) Below high school (%) Graduate (%) College degree (%)	27 (75) 4 (11.1) 3 (8.3) 2 (5.6)	8 (50) 7 (43.8) 1 (6.3) 0	0.05
Financial level*			
Low-income Intermediate	1 (5.9) 15 (88.2)	4 (2.7) 32 (86.5)	0.731
Family history of cancers	3 (8.1)	2 (11.8)	0.645
Family history of stomach cancers	2 (5.7)	1 (6.3)	0.686
High grade cancer**	17 (58.6)	3 (25)	0.05
High stage cancer***	25 (86.2)	7 (58.3)	0.06
Mortality	7 (18.6)	2 (11.8)	0.41
Vasculature prominent in the gastric tissue	17 (45.9)	9 (52.9)	0.771
CT scan positive	10 (52.6)	5 (45.5)	0.5

* 5 (one HP negative; 4 HP positives) non-reported economic levels;

** grades below grade 3 were considered low grade

*** higher than stage mean stages 3 & 4; Classification and grading of gastritis was performed using the updated Sydney System. International Workshop on the Histopathology of Gastritis, Houston [11]; staging was performed according to the recommendations of the union for international cancer control (UICC) (12, 13).

**Table 2:** Treatment modality in the study population regarding their HP histopathological result

**Type of treatment**	**Existence of Helicobacter Pylori**		**Total**
	**Negative**	**Positive**	
No treatment	2 (11.8)	4 (10.8)	6 (11.1)
Surgery alone	6 (35.3)	13 (35.1)	19 (35.2)
Surgery & chemotherapy	5 (29.4)	14 (37.8)	19 (35.2)
Chemotherapy alone	4 (23.5)	5 (13.5)	9 (16.7)
Surgery & radiotherapy & chemotherapy	0	1 (2.7)	1 (1.9)

## Discussion

H pylori is a widespread gastric pathogen which involves over half of the world’s population (14); nonetheless, fortunately, only a limited number of infected subjects will develop gastric carcinoma. The current study was conducted to evaluate the associated factors of finding H pylori infection histopathological samples of gastric tissues in our gastric cancer population. In this study, we found that in patients with gastric cancers, existence of H pylori in the gastric tissues is significantly associated with having a higher histopathological grade of the cancer. Previous studies showed that H pylori infection is associated with increased risk of gastric carcinoma (15); however, to our knowledge, the significance of finding H pylori within the gastric carcinoma tissues has not been determined. In gastric lymphomas, lodgement of H pylori within the gastric tissues was associated with low-grade MALT gastric lymphomas; but our finding evaluating gastric carcinoma showed different results with H pylori infected patients representing higher disease stage. Undoubtedly, future prospective studies are needed to confirm our results. 

Another significant finding of the present study was that detection of H pylori infection within gastric cancer tissues has been a predictor for higher stage of the malignancy. Most studies published before have analyzed seropositivity of H pylori and its associations, and even we did not find any study that focuses on its potential effects on the disease stage. So, we consider this finding as a novel finding in the literature; although future studies with larger patient population, and preferably with prospective methodology are needed.

We also found that patients with gastric cancer with either the lowest or the highest educational levels were significantly more likely to be positive for H pylori infection (table 1). The epidemiology of H pylori infection in the general population shows that people of lower educational levels are at a significantly higher risk of being infected, and the lowest rate has been reported from the highest educational level group (16), although the study was based on seroprevalence and not histopathological evaluations. On the other hand, studies investigating the rate of gastric cancers in the general populations, have not found any relation between educational status and the incidence of gastric cancer (17). We believe that this finding is also new to the literature, and needs further evaluations. But how one can explain the finding is a tough matter, especially why patients of highest educational levels had also high rates of H pylori infection. Firstly, we should consider that our whole population has been selected from gastric cancer patients. This means that also in the general population, the rate of H pylori infection in the people of higher educational level is lower, the very high level of infection in patients with gastric cancer with highest educational level may show the important role of H pylori, in producing gastric cancer. 

Female gender was another factor that significantly predicted H pylori infection in our gastric cancer patients. Epidemiological studies on the general populations show a male preponderance in the infection rate by H pylori (18, 19); although there are controversial reports representing comparable rates (20-22), we found no study that has reported a female predominance. However, our finding of a gender disparity in the rate of infection in the gastric cancer population is a novel finding, and may show that females are more vulnerable to develop gastric cancers after getting H pylori infection; this idea would be quite interesting while we know that males have shown higher risk of developing other related side effects associated with H pylori infection, including gastroesophageal reflux and duodenal ulcer development and its perforations (23-25); though prospective studies with large patient population are still needed to confirm this presumption. In this study, economic status of patients was not found to be a significant predictor of H pylori infection. Socioeconomic status is a well-defined factor that can predict H pylori infection in the general population (26-27). However, again we should emphasize that our study only includes patients with gastric cancer, and the epidemiology of H pylori infection may be quite different than the general population. Moreover, because our hospital is a military hospital where in most of the patients are from the military service, almost 90% of them rated themselves as having intermediate economic status. This decreases the credibility of this finding of our study.

This study is one of the premier studies that investigated the significance of H pylori infection in patients already diagnosed with gastric cancers. Quite new findings have been reported in the current study that adds significant data to the literature. Nevertheless, it has also some limitations. Cag A is a very significant factor that has high correlations with different aspects of gastric cancers (17). Unfortunately, we did not evaluate the status of our patients with H pylori infection regarding their Cag A antigen. Moreover, the study population was limited, and only from a single center. So, future multicentric studies with larger populations are recommended.

This study has limitations. Despite the originality of the methodology, the limited sample size may compromise the credibility of the results of the current study. Moreover, this is a retrospective study of a single referral center. Further studies with prospective approach that uses a population based study would be recommended for the confirmation of our results.

In Conclusion, the current study, we found that gastric cancer patients with H pylori are more likely to represent disease of lower grade, female gender was also another factor associated with a higher rate of H pylori detection in gastric cancer tissue. 

## Notes

**Funding:** This study has been supported by a grant from Baqiyatallah University of Medical Sciences, Iran.
